# Metabolic Dysfunction in Hutchinson–Gilford Progeria Syndrome

**DOI:** 10.3390/cells9020395

**Published:** 2020-02-08

**Authors:** Ray Kreienkamp, Susana Gonzalo

**Affiliations:** 1 Edward A. Doisy Department of Biochemistry and Molecular Biology, Saint Louis University School of Medicine, St Louis, MO 63104, USA; 2 Department of Pediatrics Residency, Washington University Medical School, St. Louis, MO 63105, USA; rkreienkamp@wustl.edu

**Keywords:** progeria, metabolism, lamins

## Abstract

Hutchinson–Gilford Progeria Syndrome (HGPS) is a segmental premature aging disease causing patient death by early teenage years from cardiovascular dysfunction. Although HGPS does not totally recapitulate normal aging, it does harbor many similarities to the normal aging process, with patients also developing cardiovascular disease, alopecia, bone and joint abnormalities, and adipose changes. It is unsurprising, then, that as physicians and scientists have searched for treatments for HGPS, they have targeted many pathways known to be involved in normal aging, including inflammation, DNA damage, epigenetic changes, and stem cell exhaustion. Although less studied at a mechanistic level, severe metabolic problems are observed in HGPS patients. Interestingly, new research in animal models of HGPS has demonstrated impressive lifespan improvements secondary to metabolic interventions. As such, further understanding metabolism, its contribution to HGPS, and its therapeutic potential has far-reaching ramifications for this disease still lacking a robust treatment strategy.

## 1. Introduction

Hutchinson–Gilford Progeria Syndrome (HGPS) is a devastating premature aging disease. Patients succumb to a variety of aging-related complications, including bone and joint abnormalities, skin changes, alopecia, and lipodystrophy [1,2]. However, their ultimate decline occurs secondary to cardiovascular complications, including left ventricle and vascular smooth muscle cell (VSMC) dysfunction, leading to death at the average age of 14.6 years [3,4,5]. The disease results mostly from a single nucleotide substitution in the *LMNA* gene, most commonly 1824C>T; G608G, wherein a cryptic splice site is activated [6,7]. Aberrant splicing of the mRNA generates a protein that lacks fifty residues, including the site of cleavage of prelamin A precursor protein to produce mature lamin A by the metalloprotease Zmpste24 or FACE1. This truncated lamin A isoform is called progerin and differs from mature lamin A in that it is permanently farnesylated and carboxyl methylated. Lamin A normally integrates seamlessly into the nuclear lamina, a proteinaceous layer just inside the inner nuclear membrane essential for proper nuclear architecture, organization of chromatin, a scaffold for various transcription factors, and normal DNA repair [8,9,10,11]. However, progerin’s permanently farnesylated tail disrupts these processes. As such, HGPS nuclei are morphologically abnormal [12,13], with blebbing and invaginations, and the function of the nucleus is severely perturbed, leading to changes in gene expression and increased levels of DNA damage [14,15,16,17,18,19]. These cellular changes drive the aging process at the organismal level.

Fortunately, HGPS is a rare disease. With an incidence of 1 in 20,000,000 births, it is estimated that there are only 400 children living with this disease in the world [20] (Progeria Research Foundation). However, understanding the molecular mechanisms underlying progerin toxicity is relevant for normal aging, since progerin is reported to be produced in aging normal individuals to some extent due to spontaneous usage of the cryptic splice site that is activated in HGPS patients [13,21]. Interestingly, a study on centenarians did not find expression of progerin [22]. These results suggest that more studies should address the functional relationship between progerin levels and healthspan and lifespan during normal human aging. It is hoped that better understanding of HGPS pathophysiology might shed light on the normal aging process and suggest avenues for slowing aging in all individuals.

Since 2003, when progerin was discovered, scientists have explored a variety of methods to counteract its detrimental effects. In spite of their efforts, the best treatment to date only extends life by 1.6 years, with significant side effects [3,4]. This drug, the farnesyltransferase inhibitor lonafarnib, works by interfering with the post-translational modification of prelamin A to progerin. Fortunately, other therapies are on the horizon. Some have focused on reducing progerin levels, whether by genetic modifications, decreasing progerin expression, or increasing its clearance. Other therapies have targeted the detrimental downstream effects of progerin. In the end, a combination of therapeutics will likely be necessary to minimize toxicity and extend healthspan and lifespan in these complex patients.

Over the years, it has become evident that progerin induces inflammation, DNA damage, epigenetic changes, and stem cell exhaustion. In addition, metabolic dysfunction continues to be viewed as an important factor in the progeria phenotype. This might be unsurprising, since metabolic dysfunction is a known contributor to normal aging [23,24]. However, the molecular mechanisms underlying metabolic alterations in progeria have been understudied. Recently, new emphasis has been placed in defining these mechanisms in progerin-expressing cells and mouse models of HGPS. In this review, we highlight some of the recent findings on metabolism, how they might contribute to in vitro and in vivo phenotypes, and how they might be exploited in future treatments for disease.

## 2. The Progeria Patient

**Figure 1:**
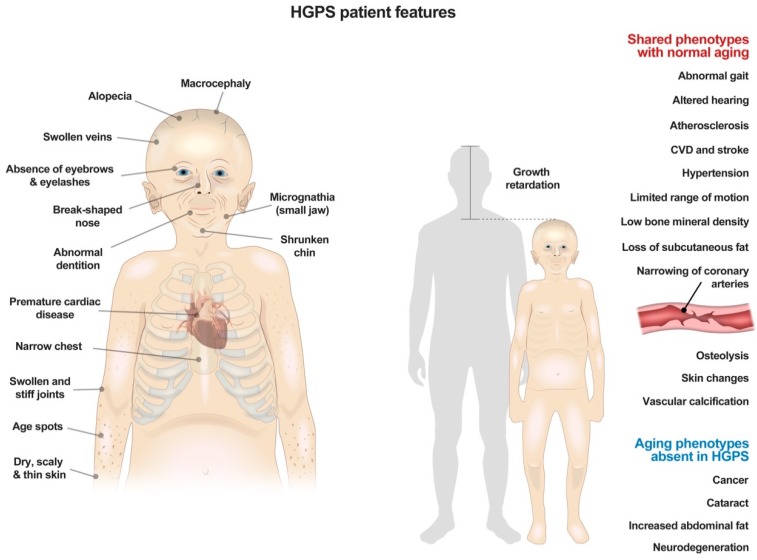
Summary of physical features and clinical symptoms in patients with Hutchinson–Gilford Progeria Syndrome.

HGPS is truly a segmental aging disease. This is because some organ systems are decimated, whereas others are unaffected. Skin and hair abnormalities, including alopecia, prominent superficial veins, and dyspigmentation, are all readily evident before 24 months of age [25] (Figure 1). Patients also develop a skeletal dysplasia, with low axial bone mineral density and distinct abnormalities in bone structural geometry and skeletal strength [26]. The most significant problems that develop are the cardiovascular complications, which ultimately lead to patient death. Patients have an impressive loss of VSMC, with associated atherosclerosis and adventitial fibrosis [27,28]. Patients also develop increased pulse-wave velocity, which is associated with vascular wall thickening and hypertension [29]. While systolic function is relatively preserved, diastolic dysfunction develops in a majority of patients, and mitral and aortic valve annular and chordal calcifications are also observed [5]. Ultimately, over 80% of deaths in HGPS result from premature, progressive atherosclerosis leading to heart failure (Progeria Research Foundation (PRF), 2019).

In spite of these severe manifestations in some body systems, other systems, like the nervous system, are spared, and patients have completely normal cognition (Figure 1). This is likely because lamin A, and hence progerin, are not highly expressed in the brain due to miR-9, a brain specific microRNA that decreases lamin A expression [30]. As such, some features of aging, like memory decline, do not occur in these patients.

Because of the gross imbalance in disease burden between different organ systems, most research has addressed the pathology associated with those systems most obviously affected. This prudent approach has yielded a much better understanding of cardiovascular pathology, as well as other major contributors to progerin-induced disease. However, other properties, like metabolism, have not been more extensively studied until recently, and there is much still to learn.

From afar, it would seem that these patients have some metabolic alterations. Often an initial manifestation of disease is failure to thrive, usually before one year of age [1,2]. Patients are distinctively smaller than their peers, and most never reach 30 kg [31] (Figure 1). They gain weight at a decreased and linear rate, greater than two standard deviations below age-matched peers [32]. This is in spite of the fact that HGPS patients consume enough food to grow, but just do not because of their disease (Progeria Research Foundation (PRF), 2019).

Fat stores are also significantly decreased in HGPS patients, and the proportion of lean body mass to less metabolically active fatty tissue is significantly increased in HGPS [5]. Decreased fat stores and adipose tissue has numerous systemic effects beyond those readily apparent. At the macroscopic level, lack of fat causes functional consequences, such as foot pain due to lack of subcutaneous fat under the calcaneus. At the microscopic level, many important signaling markers are altered. Leptin, which is produced in adipose tissue and helps to regulate energy expenditure, is significantly decreased in HGPS patients [33]. This is significant since low leptin levels have previously been shown to suppress energy expenditure and immunity, and, in some cases, have also been linked to cardiovascular disease [34,35]. Remarkably, HGPS patients do not seem to have alterations in the immune system, responding well to infections and healing wounds normally [36]. Insulin is significantly increased in HGPS, rising from 2.56 μIU/mL in controls to 9.03 μIU/mL in HGPS patients [33]. Some patients also develop insulin resistance and require treatment for diabetes, though most do not. It is well-established that, even without diabetes, insulin resistance is a marker of cardiovascular risk [37,38]. Therefore, this could be a contributor risk in these patients. Additionally, many other endocrine proteins are altered in HGPS, including adiponectin [33]. While some of these are corrected by treatment with FTIs, many still remain altered after treatment, suggesting a window for other combination therapies [33].

Fat stores are also known to be linked with sexual hormone expression. Very little has been studied regarding pubertal development in these patients. However, a recent study showed that while some girls do enter menarche, most do so without passing Tanner stage II sexual development [39]. More studies are needed to determine if decreased levels of estradiol prevent full sexual development. Given the importance of estrogen to cardiovascular health in women [40], understanding these parameters have far reaching ramifications.

While some endocrine factors have been studied in these patients, there are still big gaps in knowledge about metabolism and endocrinology in HGPS. Studying these parameters, and finding targetable deficiencies, might hold promise for the cardiovascular disease and other important phenotypes of these patients, which to this point remain incompletely linked to these known endocrine alterations.

## 3. The Progeria Cell and Energy

The basis for HGPS organismal changes lies at the cellular level. It is evident that there are anomalies in energy generation and expenditure, mostly stemming from alterations in mitochondrial function [41,42] and in signaling pathways regulating anabolic and catabolic processes in response to cues from the environment [43]. The mechanistic target of rapamycin (mTOR) protein complexes -mTORC1 and mTORC2-, regulated by AMPK and AKT kinases, sense the levels of nutrients and energy in cells and execute signals to either synthesize macromolecules when sources of energy and nutrients are high, or to activate autophagy in the context of starvation [44,45]. There are many examples of mTOR inhibition increasing lifespan in diverse organisms. Reducing mTOR activity increases autophagic flux, reduces reactive oxygen species (ROS) and increases cellular replicative lifespan. Importantly, mTOR regulates mitochondrial function via different mechanisms, including the modulation of transcription and translation of important mitochondrial genes [46].

In 2011, the finding that rapamycin treatment of HGPS patient-derived fibroblasts slows cellular decline and enhances progerin clearance generated much excitement [47]. The beneficial effect of rapamycin is potentiated by including all-trans retinoic acid (ATRA) in the treatment [48]. In addition to improving the phenotypes of HGPS fibroblasts, rapamycin rescues age-related changes in muscle-derived stem/progenitor cells (MDSPCs) from progeroid mice. In particular, MDSPCs isolated from prelamin A-expressing progeria mice exhibit increased mTOR signaling. This is accompanied by proliferation and differentiation deficiencies in culture and during tissue regeneration [49]. Inhibition of mTOR with rapamycin improved differentiation along myogenic and chondrogenic lineages and reduced the extent of apoptosis and senescence of MDSPCs. The same results were obtained in another progeria model caused by genomic instability due to reduced levels of the nuclease ERCC1-XPF [50]. Perhaps the clearest example of rapamycin benefits for laminopathies is the effect on lamin A/C-deficient mice. Cardiac and skeletal muscle from these mice show elevated mTOR signaling and rapamycin improves muscle function and mice survival [51]. All these studies highlight the relevance of mTOR as a potential therapeutic target for improving the health of aged stem and somatic cells. However, there is no consensus in the field yet about the activation of mTOR signaling in progeria cells of different origin and about the net beneficial effects of rapamycin treatment in different mouse models of HGPS. Some investigations for instance, did not find evidence for mTOR pathway activation in HGPS cultures [47,52]. In fact, reduced mTOR activity has been reported in some studies and associated with increased basal levels of autophagy [53]. Nevertheless, the benefits of rapamycin are considered for the most part to outweigh its associated toxicity, and thus rapalogs have been included in HGPS clinical trials (discussed below).

Mitochondrial dysfunction is a hallmark of HGPS patient-derived fibroblasts and mouse models. Initial studies reported a marked downregulation of mitochondrial oxidative phosphorylation proteins and reduced ATP production and pyrophosphate levels, concomitant with upregulation of glycolytic enzymes [41,54,55]. In particular, fibroblasts from the patients show markedly lower expression of three crucial components of the mitochondrial respiratory chain, including cytochrome c, the complex IV component cytochrome c oxidase subunit I, and the complex V protein -ATPase [41]. The decrease in ATP content was linked to increased protein oxidation and protein damage with cellular passage [56]. In addition, progerin expression causes an increase in swollen and fragmented mitochondria, and decreased mitochondrial movement, possibly related to reduced availability of cellular ATP for proper microtubule-mediated transport [57]. Mitochondrial dysfunction seems to be critical for the excessive vascular calcification and the loss of vascular smooth muscle cells (VSMC) that characterizes HGPS [54].

Decreasing mitochondrial dysfunction markedly improves cellular phenotypes. Treating HGPS cells with methylene blue, a mitochondrial-targeting antioxidant, alleviates mitochondrial defects and reduces nuclear abnormalities, perinuclear heterochromatin loss, and misregulated gene expression [57]. Progerin also hinders antioxidant mechanisms that counteract the accumulation of ROS caused by oxidative phosphorylation. For instance, progerin sequesters Nuclear factor erythroid 2-related factor 2 (NRF2) NRF2, a master regulator of antioxidant gene programs, causing subnuclear mislocalization and chronic oxidative stress, DNA damage, and increased progerin levels [58]. Restoring proper NRF2 activity greatly ameliorates HGPS cellular phenotype by reinstating this normal antioxidant pathway. Importantly, NRF2 pathway activation rescues the viability of HGPS mesenchymal stem cells (MSCs) in vivo [58], implying that this pathway could be targeted to retard the exhaustion of adult stem cell populations that occurs in HGPS. Few other therapies, to this point, have targeted mitochondrial dysfunction in ameliorating aging. Some current therapies have even been noted not to affect mitochondrial dysfunction. For instance, temsirolimus, a rapamycin analog which improves many other cellular phenotypes, does not improve mitochondrial function [42]. Thus, this represents an area ripe for novel therapies for treating disease. Further research in this area could identify novel targetable pathways to improve disease phenotype.

Another important family of proteins regulating cellular energy levels and survival under stress conditions is the sirtuin family, proteins with NAD-dependent deacetylase and ADP-ribosyltransferase activities that also participate in DNA repair [59]. SIRT1 for instance, activates AMPK and enhances metabolic efficiency, among other roles [60]. SIRT6 regulates glucose homeostasis and inflammatory signaling through histone deacetylation at multiple genes involved in metabolism [61]. Importantly, SIRT1 activity decreases with age and treatment with the SIRT1 activator resveratrol improves aging pathologies in progeria mice, including adult stem cell decline [62]. Similarly, SIRT6 depletion causes premature aging and shortening of lifespan [63]. SIRT6 expression is downregulated in HGPS cells and SIRT-6 overexpression ameliorates senescence phenotypes in vitro [64] and extends lifespan of wild-type male mice. Thus, sirtuins are emerging as important anti-aging targets.

Elegant studies recently uncovered crosstalk between the metabolic pathways discussed above and the DNA damage response (DDR). In particular, the critical DNA damage sensor protein ATM has been shown to functionally decline during cellular aging/senescence and ATM knockout mice show premature aging phenotypes [65]. During senescence, DDR deficiencies are accompanied by changes in glucose metabolism characterized by impaired mitochondrial respiration and increased glycolysis, producing more lactate [66]. Activation of ATM via a low dose of chloroquine, an autophagy inhibitor that is used to treat malaria, results not only in improved DNA damage clearance, but also rescue of age-related metabolic changes (inhibition of glycolysis) and prolonged cellular replicative capacity [67]. Mechanistically, ATM phosphorylates SIRT6, which leads to protein stabilization and enhanced function. Studies in vivo show that extra copies of Sirt6 in ATM-deficient mice restore metabolic homeostasis and extend lifespan. Given that progeria cells exhibit deficiencies in ATM and SIRT6 function, the authors investigated the possibility of using chloroquine to improve aging phenotypes in vitro and in vivo [67]. Relevantly, chloroquine treatment of progeria cells activated Atm, stabilized Sirt6, reduced DNA damage, inhibited glycolysis, and ameliorated senescence. Chloroquine treatment of progeria mice delayed body weight loss, enhanced endurance, and prolonged lifespan. These studies reveal the Atm-Sirt6 axis as a new pathway modulating DNA damage, metabolism, aging and longevity.

In summary, our understanding of metabolism in cells from HGPS patients and from mouse models of the disease is very limited. Classical pathways associated with metabolic alterations in other diseases are beginning to be interrogated in progeria, including mTOR, AMPK, Sirtuins, mitochondrial function, and most recently ATM signaling. However, significantly more effort is needed in years to come to define the impact of progerin expression on metabolic pathways and the contribution of metabolic reprogramming to loss of tissue homeostasis and accelerated aging.

## 4. The Progeria Mice

The majority of work on metabolism in HGPS, to this point, has been done in mouse models of the disease. All models with alterations in lamins function have shown metabolic changes to some extent. Initially, scientists generated the *Lmna^-/-^* mouse model [68], which was developed to be lamin A and C null. Mice acquire significant lipodystrophy and almost complete absence of white fat. Growth is severely retarded as early as two weeks of age, and most mice die by eight weeks of age. Next, two different *Zmpste24^-/-^* mouse models, lacking the essential cleavage enzyme in prelamin A processing and generating prelamin A, were developed, where mice also had decreased weight, lipodystrophy, and cachexia [69,70]. These mice were also noted to foster hypoglycemia before death, which occurred around 20 weeks of age. In 2006, a transgenic model of HGPS was generated that carried a human bacterial artificial chromosome harboring the common human mutation. This model recapitulated the progressive vascular smooth muscle cell defects characteristic of HGPS patients [71]. Finally, in 2011, the *Lmna^G609G^* mouse model, which carried the main mutation in the *LMNA* gene driving HGPS in humans, was generated [72]. These mice, particularly the homozygotes, have shortened lifespan, most dying around 100 days of age on a regular chow diet. These mice also become hypoglycemic before death, with distinctive alterations in body fat and energy expenditure. Although humans are generally heterozygote, most studies are done in homozygous mice, as these better recapitulate the severity of the human disease.

Since the *Lmna^G609G^* mouse model best recapitulated the human phenotype with an equivalent mutation to that seen in humans, researchers have focused most of their work in this model over the last decade. Initially, it was found that mitochondrial DNA was significantly increased in these mice [55]. This was accompanied by increased basal oxygen consumption rate and increased basal ATP production. These changes could be responsible for increased oxidative stress in these mice. However, this study focused mostly on heterozygotes mice, and the result might vary with the mutation in homozygosis.

The *Lmna^G609G^* mouse model also develops vascular disease, with loss of VSMC and excessive vascular calcification [54,72]. The etiology for this cardiovascular disease has been an area of intense research due to the importance of cardiovascular disease to human phenotype. The excessive vascular calcification in these mice, which recapitulates the human phenotype, results at least in part from reduced extracellular pyrophosphate accumulation, stemming from diminished ATP availability due to mitochondrial dysfunction [54]. Mitochondrial dysfunction is thought to be a significant driver of the VSMC dysfunction. Recently, nitrite supplementation, which protects cells against oxidative stress and mitochondrial dysfunction, was found to decrease VSMC stiffness [73]. More work is needed to explore further the cellular mechanisms driving VSMC death, and especially as it relates to mitochondrial dysfunction.

For the *Lmna^G609G^* mouse model as a whole, initially researchers supported the thought that these mice were dying from cardiovascular complications, just like their human counterparts. However, these mice did not develop typical atherosclerosis like in humans, in spite of VSMC loss. Lack of atherosclerosis is not totally surprising, since mice are known to be resistant to atherosclerosis given their high HDL profile [74]. These mice also did not show any post-mortem signs of hemorrhage, stroke, or myocardial infarction. More impressive at autopsy was the loss of fat and muscle. Even when these mice were crossed with the ApoE mice, they still died at an early time point, and their lifespan was unaffected by treatments designed to improve cardiovascular health [75]. When a VSMC-specific progerin-expressing mouse model was generated, these mice died at much later times than the ubiquitously progerin-expressing mice, despite VSMC loss being similar between both models at equal time points [76]. Interestingly, mice in which progerin expression is targeted selectively to endothelial cells develop interstitial myocardial and perivascular fibrosis, and left ventricular hypertrophy and diastolic dysfunction, but not VSMC loss [77]. Approximately 40% of these mice experience sudden death when approaching 25 weeks of age, which could fit the idea of myocardial infarction. Altogether, these findings suggest that defects in the endothelium and in smooth muscle cells upon progerin expression cooperate causing cardiovascular disease (CVD). Whether it is CVD or something outside the vasculature that is driving *Lmna^G609G^* mouse death needs further investigation.

## 5. Metabolic Approaches in Progeria

Some researchers had proposed that *Lmna^G609G^* mice die because of cardiac arrhythmias. There is some merit to this idea, especially given the increasing QRS interval that normally precedes these mice death [72]. However, these electrical alterations presented around the time when these mice developed signs of hypoglycemia. It has been well established that hypoglycemia can lead to cardiac arrhythmias. In fact, previous studies have shown that hypoglycemia can increase the QTc interval [78,79]. Therefore, even with this hypothesis, the metabolic dysfunction could be the driving force.

**Figure 2:**
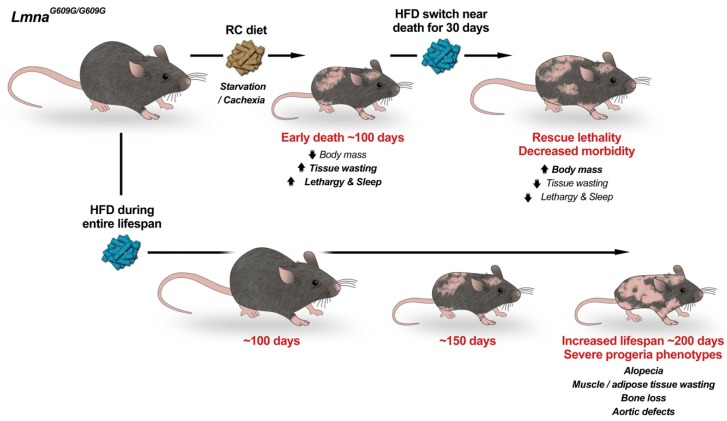
High Fat Diet doubles the lifespan of progeria mice. Progeria *Lmna^G609G/G609G^* mice fed a regular chow (RC) diet die from starvation and cachexia at ~100 days of age (top). Switching progeria mice approaching death from RC to high-fat diet (HFD) rescues early lethality and decreases morbidity. Importantly, feeding only HFD (bottom) delays aging and nearly doubles lifespan. During their extended lifespan progeria mice develop degenerative aging pathologies of a severity that mirrors the human disease. We propose that metabolic alterations greatly influence progeria phenotypes and that nutritional/nutraceutical strategies might help modulate disease progression. Progeria mice on HFD represent a more clinically relevant animal model to study mechanisms of HGPS pathophysiology and to test therapies.

Given this hypoglycemia and bradykinesia which preceded death, our lab began to embark on a more thorough metabolic characterization of these mice [80,81]. Interestingly, *Lmna^G609G/G609G^* mice have energy alterations well before their ultimate decline. Differences in mass develop by four weeks, as *Lmna^G609G/G609G^* mice are significantly smaller than their wild-type counterparts from that point forward. Mice also conserved energy to a greater degree than their wild-type peers, running less than their wild-type counterparts when placed in wheel running cages. Lean mass began to decrease by seven weeks of age, and fat mass was also significantly decreased. *Lmna^G609G/G609G^* mice absorbed more food than their wild-type counterparts, potentially because of the greater need for energy due to deficiencies in energy generation. As death approached, mice were barely capable of moving and were noted to be hypoglycemic. To determine if a more energy-rich diet might extend mouse lifespan, we placed *Lmna^G609G/G609G^* mice nearing death on a high-fat diet (HFD) (Figure 2). Remarkably, these mice regained some of their mass and lived for at least an additional month. Because of this, we wondered what would happen if mice were placed on HFD earlier in life, to prevent the early decline in lean and fat mass. While some scientists have previously placed these mice on HFD for some weeks [76], none had previously placed these mice on HFD since birth. And, impressively, the effect was greatest with this strategy. Mice fed this HFD gained weight more than their peers on regular chow, reaching an average maximum mass of 20.3 g, 14% above their regular chow counterparts at nine weeks, and they had increased fat mass (Figure 2). Also, mass began declining at a later time point for mice on HFD. While other progeroid phenotypes became more evident, such as alopecia, VSMC loss, and bone abnormalities as these mice aged, the mice thrived and had the greatest lifespan extension to date (Table 1), extending lifespan by 71.7%, in a mouse model of HGPS (Figure 2). Importantly, female mice supplemented with high-fat diet also were able to become pregnant and deliver litters, a feat previously unattainable in these mice. This indicates that energy stores contribute to the fertility of these animals. Thus, these studies were revolutionary in that they revealed the impact of progerin expression on metabolism in vivo and its relevance for global disease phenotype.

Given the impressive benefit of the HFD in this mouse model, the finding that methionine restriction (MR), a mimicker of calorie restriction, also extended lifespan seemed puzzling [84]. There, methionine restriction, starting at seven weeks of age in *Lmna^G609G/G609G^* mice, increased median lifespan by 20%. Previous studies have shown that methionine restriction did extend lifespan in other mouse models [88]. The finding that methionine restriction, a calorie restriction mimicker, and HFD both extend lifespan are not mutually exclusive. More attention is needed to understand these diets as a whole and determine those dietary interventions most crucial to extending lifespan. Even the timing of when to initiate a dietary intervention can have profound effects on outcome. For instance, previous studies in another mouse model showed that intermittent fasting did not increase lifespan when started at six months of age, but did increase mean lifespan by 15% when started at 10 months of age [89]. Between these two studies, there are numerous differences in the approach to these dietary interventions. The HFD was initiated at birth and seemed to have its largest benefit before nine weeks of age. On the other hand, methionine restriction was begun at eight weeks of age. Therefore, further characterization of the mechanisms behind these diets might lead to better understandings of how diets might impact disease phenotype. Regardless, these studies are extremely exciting in that they reveal that the relatively benign prospect of altering diets could have profound consequences on the healthspan and lifespan in progerin-expressing organisms.

In clinical circles, metformin has become the latest rage in the field of aging. While metformin has previously been used for years as a first-line treatment for type II diabetes due to its ability to decrease hepatic glucose output, recent research has also demonstrated that metformin decreases insulin levels, reduces endogenous production of reactive oxygen species (ROS), activates AMPK, and reduces DNA damage [90]. As such, metformin is now being considered as a tool for cancer and other aging-associated diseases [91], and the TAME trial will determine if in fact metformin can slow aging in humans. With this in mind, it seems natural that metformin might benefit HGPS patients, where patients are known to have hyperinsulinemia, increased ROS, and increased DNA damage. Interestingly, when HGPS dermal fibroblasts were treated with metformin, there was partial restoration of normal nuclear phenotypes, delayed senescence, decreased DNA damage, activation of AMPK phosphorylation, and decreased ROS formation, all while also suppressing progerin expression [92]. Another group also showed the benefit of metformin, though suggested this might be through decreased SRSF1 expression, which has been shown to affect alternative splicing of *LMNA* in humans and mice [93]. Thus, as we understand more about progeria and metformin, this is one such drug which has important metabolic effects which might gain greater importance in the coming years.

## 6. Current Therapeutic Strategies

With as many organ systems and cellular processes that are affected in HGPS, it is likely that a combination of therapies will be needed to treat HGPS. Fortunately, a number of therapies have been developed in the last five years which might help patients. Three main areas have been targeted in developing therapies to treat disease: (1) Preventing full post-translational processing to progerin (2) Decreasing progerin production and (3) Ameliorating deleterious downstream effects of progerin.

As discussed, farnesyltransferase inhibitors are the only compound to have shown benefit in patients thus far, extending lifespan by about 1.6 years [94]. These work by preventing farnesylation of progerin. Lonafarnib was first used in vitro and then in vivo with significant success [95,96,97,98,99]. Other post-translational modification blockades have been attempted, but none have had as robust of an effect. Isoprenylcysteine carboxyl methyltransferase (ICMT) inhibition, which inhibits carboxymethylation during prelamin A processing and disrupts proper targeting of CAAX protein to cell membranes, showed promise in in vivo models of disease, but no pharmacological inhibitors have yet been used in vivo [100].

Next, researchers have begun exploring ways to decrease levels of progerin. The closest to widespread clinical use is everolimus, a rapamycin analog, which works by increasing the clearance of progerin. In cellular models, both rapamycin [47] and everolimus [101] caused a remarkable improvement in cell phenotype. A current clinic trial (ClinicalTrials.gov Identifier: NCT02579044) is in stage two and is testing the effectiveness of lonafarnib and everolimus in combination. Primary completion is expected in December 2020. Other compounds have shown promise in preclinical trials to decrease levels of progerin. These include all-trans retinoic acid [48], sulforaphane [102,103], and calcitriol (the active, hormonal form of vitamin D) [104]. All now need to be tested in mouse models of disease to determine if they can produce similar effects in vivo.

The advent of CRISPR-therapies has sparked new hope for genetic approaches to reduce progerin production. HGPS is a perfect disease to treat by genetic approach, since correcting a single-point mutation drastically improves disease. Originally, it was shown in *Lmna^G609G^* mice that antisense morpholino–based therapy preventing pathogenic *Lmna* splicing markedly improved mouse phenotype and extended lifespan [72]. In a different study, an antisense oligonucleotide (ASO) was identified that increased lamin C production while reducing progerin [105]. When this ASO was administered to mice, a reduction in progerin expression was observed in murine tissues, highlighting the potential therapeutic use of ASOs in HGPS. Recently, for the first time, two papers have shown the benefit of CRISPR-therapies in vivo [85,86]. Injecting gRNAs into HGPS-Cas9 mice, via facial vein or intraperitoneally, both significantly increase lifespan of progeroid mice. This is in spite of the fact that uptake was relatively poor. Thus, as this technology becomes more refined, it is likely that these therapies will become even more successful.

Other compounds have targeted cellular pathways disrupted by progerin production. One of the most striking compounds has been remodelin, which improves nuclear architecture, chromatin organization, and fitness of progerin-expressing cells, without significantly impacting progerin production [106]. Targeting NAT10, the target of remodelin, also significantly increase lifespan in a mouse model of disease [83]. Other novel therapies, like in vivo partial reprogramming, also improved the phenotype [82].

Perhaps one of the most intriguing and exciting recent discoveries about progeria treatment is the use of fecal microbiota transplantation. Barcena el al. reported intestinal dysbiosis -increased abundance of Proteobacteria and Cyanobacteria and decreased abundance of Verrucomicrobia- in two progeria mouse models and in HGPS patients. Interestingly, transplantation of fecal microbiota from wild-type mice to progeria mice improved healthspan and lifespan. These results open the door for new microbiome-based therapeutic strategies for HGPS [87].

Therefore, there exists a number of therapies, targeting many different cellular pathways, that could greatly improve HGPS cell phenotype. Over the coming years, many of these will be further explored to determine if they can synergize with current therapies for disease.

## 7. A Case for Metabolism

Much effort has been invested in finding the new greatest tool to extend healthspan and lifespan in progeria. And, the prospect for treatments are now greater than at any point in the last twenty years, with a myriad of therapies nearing testing in humans. As technology expands, the hope for extending healthspan and lifespan of these children will grow even further.

In spite of this, recent studies in mouse models have shown that dietary interventions increase lifespan more than any other recorded therapy to date. Future studies are now needed to determine what specific dietary components lead to the marked increase in lifespan.

Over the years, a variety of diets have been proposed to help with normal aging. Recently, the ketogenic diet and intermittent fasting have garnered increased attention [107,108]. But will those work in HGPS? Likewise, can giving extra calories, as was done in high-fat diet, particularly early on as these kids are growing, increase longevity for these children? When should these diets be initiated?

While these mouse models have shown promise, translating metabolic studies in mice to humans can be challenging. This is because mice have a specific metabolic rate ~7× greater than humans and have differences in mitochondrial density [109]. Further, in these mice, it seems that metabolic dysfunction is what drives their death, not atherosclerosis as in humans. Therefore, any dietary approaches attempted in humans must be certain not to worsen atherosclerosis and cardiovascular disease. Yet, some who have cared for these patients suspect that those patients who have meticulously watched their diets have had better outcomes. Better logging dietary habits of patients and correlating them with outcomes might provide a better understanding for future dietary guidance.

At the cellular level, there is no debate concerning the presence of metabolic and mitochondrial dysfunction. This mitochondrial dysfunction has a significant impact on many cells within the body and could potentially be responsible for VSMC loss that is so pronounced in progeria [110,111]. Further, previous research has shown that HGPS patients are resistant to cancer due to the actions of BRD4, which inhibits tumorigenic potential of transformed cells [112]. However, the decreased ability of progerin-expressing cells to be transformed might also be related to mitochondrial dysfunction. Future studies are needed to assess this, as well as the contribution of mitochondrial dysfunction to so many other phenotypes in progeria.

In the end, metabolic dysfunction, though not seemingly the most important cause of disease in HGPS, could be a major player in disease phenotype. While we continue to invest in developing novel drugs to combat this terrible disease, understanding lifestyle factors that increase longevity are also vitally important. These might provide a more benign and effective starting point for future adjunctive therapies for disease.

## Figures and Tables

**Table 1:** Therapeutic treatments and their effect in *Lmna^G609G/G609G^* mice.

Treatment	Median Lifespan Untreated	Median Lifespan Treated	Percentage Increase	Max Lifespan	Citation
Antisense Oligonucleotides Against Progerin	111 (Mean)	155 (Mean)	39.6%	190	(Osorio et al., 2011) [72]
Partial Reprogramming	126 *	168 *	33.3% *	205 *	(Ocampo et al., 2016) [82]
Remodelin	80 *	110 *	37.5% *	120 *	(Balmus et al., 2018) [83]
Methionine Restriction	155 *	180 *	16.1% *	225 *	(Bárcena et al., 2018) [84]
High-Fat Diet	113	194	71.7%	229	(Kreienkamp et al., 2019) [80]
CRISPR-Cas9	128 (Mean)	167 (Mean)	30.4%	212	(Beyret et al., 2019) [85]
CRISPR-Cas9	140 *	177 *	26.4%	224 *	(Santiago-fernández et al., 2019) [86]
Fecal Microbiota Transplantation	141	160	13.5%	184	(Bárcena et al., 2019) [87]

* Indicates approximate days of life, since exact point was not reported in publication.
